# Managing the low carbon transition pathways through solid waste electricity

**DOI:** 10.1038/s41598-024-56167-2

**Published:** 2024-03-06

**Authors:** Muhammad Amir Raza, M. M. Aman, Ghulam Abbas, Shakir Ali Soomro, Amr Yousef, Ezzeddine Touti, Nayyar Hussain Mirjat, Mohammad Huzaifa Ahmed Khan

**Affiliations:** 1 Department of Electrical Engineering, Mehran University of Engineering and Technology, SZAB Campus, Khairpur Mir’s, 66020 Sindh Pakistan; 2 Centre for Advanced Studies in Renewable Energy (ASURE), NED University of Engineering and Technology, Karachi, 75270 Sindh Pakistan; 3 School of Electrical Engineering, Southeast University, Nanjing, 210096 China; 4 Electrical Engineering Department, University of Business and Technology, Ar Rawdah, 23435 Jeddah, Saudi Arabia; 5 Engineering Mathematics Department, Alexandria University, Lotfy El-Sied St. Off Gamal Abd El-Naser, Alexandria, 11432 Egypt; 6 Department of Electrical Engineering, College of Engineering, Northern Border University, Arar, 91431, Saudi Arabia; 7 Department of Electrical Engineering, Higher Institute of Applied Sciences and Technology of Kasserine, University of Kairouan, 3100, Kairouan, Tunisia; 8 Department of Electrical Engineering, Mehran University of Engineering and Technology, Jamshoro, 76060 Sindh Pakistan; 9 Department of Electronics Engineering, NED University of Engineering and Technology, Karachi, 75270 Sindh Pakistan

**Keywords:** Solid waste, Energy production, Capital cost, Carbon emissions, And climate system, Engineering, Electrical and electronic engineering, Energy infrastructure

## Abstract

The potential of solid waste as an energy source is clear, owing to its wide availability and renewable properties, which provide a critical answer for energy security. This can be especially effective in reducing the environmental impact of fossil fuels. Countries that rely heavily on coal should examine alternatives such as electricity from solid waste to provide a constant energy supply while also contributing to atmospheric restoration. In this regards, Low Emissions Analysis Platform (LEAP) is used for simulation the entire energy system in Pakistan and forecasted its capital cost and future CO_2_ emissions in relation to the use of renewable and fossil fuel resources under the different growth rates of solid waste projects like 20%, 30% and 40% for the study period 2023–2053. The results revealed that, 1402.97 TWh units of energy are generated to meet the total energy demand of 1193.93 TWh until 2053. The share of solid waste based electricity in total energy mix is increasing from a mere 0.81% in 2023 to around 9.44% by 2053 under the 20% growth rate, which then increase to 39.67% by 2053 under the 30% growth rate and further increases to 78.33% by 2053 under the 40% growth rate. It is suggested that 40% growth rate for solid waste based electricity projects is suitable for Pakistan until 2053 because under this condition, renewable sources contributes 95.2% and fossil fuels contributed 4.47% in the total energy mix of Pakistan. Hence, CO_2_ emissions are reduced from 148.26 million metric tons to 35.46 million metric tons until 2053 but capital cost is increased from 13.23 b$ in 2023 to 363.11 b$ by 2053.

## Introduction

In the previous few of decades, Pakistan's industrial and economic progress has been hampered by a lack of energy^1^. The country's energy shortage has prompted several large firms to cut production and employee numbers, resulting in a surge of inflation and unemployment. To meet the energy demand, desperate measures have been attempted, resulting in a transition to thermal power facilities using diesel and heavy fuel oil that are funded and run by independent power producers^2^. In 2014, Pakistan invested more than 36% of its allocation for imports on fuel purchases^3^. From 2005 to 2015, ratio of CO_2_ emissions in Pakistan to global grew from 138 million metric tons per year to 177.43 million metric tons annually as a result of the growing use of fossil energy for the production of energy^4^. Per capita CO_2_ emissions will increase significantly during the upcoming ten years as additional coal and gas fired power plants come online. Pakistan's yearly per capita CO_2_ emissions (0.8 metric tons per capita) are still significantly below than global emissions (4.996 metric tons per capita), North American emissions (16.1 metric tons per capita), and the emissions of OECD members (9.7 metric tons per capita)^5^. Power stations that burn coal and gas are a preferable alternative in the foreseeable future because of the demand for affordable electricity. The CO_2_ emissions will pose major environmental risks, however, nuclear and renewable energy facilities are clean, but due to financial and technological constraints, they are not commercially viable in a developing country like Pakistan^6^.

By partially replacing coal with solid waste as a fuel and applying sophisticated solid fuel combustion technology, this difficult circumstance can be turned into an opportunity. This will necessitate the construction of solid waste generating plants near the country's rubbish dumps. Utilizing advanced and trustworthy steam power plant technologies, this technique will then supply green electricity just by swapping out a tiny bit of coal from a solid waste. So, overall CO_2_ emissions from coal fired power stations can be reduced in a co-fired steam power plant^7^. Pakistan's energy policy needs to be adjusted and altered in order to make room for the usage of cleaner and local supplies^8^. Solid waste-fired power plants can help reduce CO_2_ emissions by up to 65 point margin, although they are not generally carbon–neutral^9^. Furthermore, Pakistan's energy deficit can be decreased in an environmentally sustainable manner by utilizing solid waste resources^10^. Power generation from biomass or solid waste is widely used around the world, with installed capacity of roughly 8140 MW, 4788 MW, 4024 MW, and 3785 MW in the United States, China, Brazil, and Japan, respectively^11^. Solid waste's low energy content and bulk density, as well as seasonal availability and pricing of feedstock, are the main factors that influence its utilization as a power plant fuel. The aforementioned parameters have a complex and non-linear impact on solid waste supply and demand^12^. This necessitates the creation by the local government of an effective strategy for using solid waste as a reliable fuel in co-fired and solid waste-only power plants^13^. The use of solid waste for manufacturing of paper, packaging materials, steam, and the possibility for it to replace fossil fuels in the production of polymers may have an impact on the supply of agricultural cellulosic biomass for electricity generation^14^.

The National Electric Power Regulatory Authority (NEPRA) in Pakistan provides a comprehensive breakdown of energy costs and tariffs. They find that power generated from solid waste is comparable in cost to fossil fuel-based power sources and is even less expensive than solar and wind energy^15^. The tariff set by NEPRA for energy derived from solid waste approximates to 8.28 US cents per kWh, while the rates for coal, natural gas, wind, and solar power are 7.79, 12.44, 8.20, and 11.31 US cents per kWh, respectively^16^. Although the operational and upfront costs of a power plant fueled by solid waste may be slightly more than those of a coal power plant, but they are significantly less than those associated with wind or solar power plants^17^. Power plants utilizing solid waste are a feasible solution for managing base energy demand and have the additional benefit of emitting less CO_2_ due to their high capacity of over 80%^18^. Presently, there exists an array of systems worldwide for steam production, pollution regulation, biogas creation, and power generation, which utilize either solid waste alone or a co-firing method^19^. Techniques like fluidized bed combustion (FBC) and chemical looping combustion systems have been successful in enhancing conversion efficiency and lowering emissions of pollutants^20^. Economic activity in developing countries results in the highest output of solid waste, which has significant environmental impacts. This source, on the other hand, might be used to generate electrical energy, bio-oil, and biofuel. As a result, solid waste utilization is advantageous because this resource is available in abundant quantity and ideal for power generation. However, the type of material and quantity of trash generated differs by region^21^.

Pakistan generates around 55,422 million tons of waste/year followed by china 850 million tons of waste/year, India 780 million tons of waste/year, Brazil 597 million tons of waste/year and European countries produces million 205 tons of waste/year^22^. The everyday production of solid waste is around 64,000 tons, which is suitable for power generation due to its high calorific value (6.9 J/kg). The entire production capacity of solid waste in the major municipalities is estimated to be around 712 million tons per year^23^. In India, the intermediate and densely populated cities produced a considerable amount of garbage, which is growing at a pace of 7.5% from 2021 to 2026^24^. Globally, agriculture sector produces more waste including sorghum (62 million tons/year), wheat (706 million tons/year), oat (23 million tons/year), barley (142 million tons/year), rice (473 million tons/year), corn (963 million tons/year), and sugarcane (1741 million tons/year)^25^.

Based on the availability of solid waste resource in Pakistan, technological and economic energy generation potential from 2023 to 2053 were assessed using the LEAP software and the utilization of solid waste as a feedstock, such as agricultural waste, municipal solid waste, industrial waste, and hazardous waste was examined. Building power plants based on solid waste resource is essential around the world and also in rural and urban sites of Pakistan and it is necessary to improve commercial-scale electricity generation from solid waste^26^. This study set out to explore Pakistan's potential for producing renewable energy from solid waste and estimating the carbon emissions and capital investment cost from 2023 to 2050. Three estimates have been considered in forecasting like 20%, 30% and 40% share of solid waste based electricity for sustainable energy mix and forecasting the future carbon emissions and total capital investment cost accordingly for implementing low carbon transition pathways for Pakistan.

This study is structured into six (06) sections. Section “Solid waste classifications” presented the existing literature on the many types of solid waste, as well as their generation, administration, and disposal techniques is briefly summarized in “Solid waste production, management and utilization”. Section “Materials and methods” covers the research method and data analysis for LEAP model. Section “Results and discussion” provides in-depth analysis of empirical results and finally conclusion is given in “Conclusion and policy implications”.

## Solid waste classifications

The majority of solid waste is generated by industries like agriculture, construction, residential housing, and commercial business^24^. The four main categories of solid waste are hazardous waste, industrial waste, municipal solid waste, and agricultural waste. Below, each waste category is covered in more detail.

### Agriculture wastes

Wastes produced as a result of various agricultural activities which are referred to as biomass waste. Agricultural wastes include things like animal faeces, post-harvest rubbish like rice husks, rotting or subpar fruits and vegetables, maize stover and husks, and wheat straw^27^. The two primary types of residues generated by agricultural activities are field residues and process residues. After reaping a crop, field wastes such branches, grains, stalks, and leaves are often left behind. On the other side, crop remnants such as roots, peel, stubble, pulp, shell, stalk, straw, stem, leaves, seeds, bagasse, husks, molasses, and other processing remnants are signs that the crop has been transformed into valuable substitute commodities^28^. Depending on their availability and characteristics, agricultural waste can be distinguished from other solid fuels including charcoal, wood, and char briquettes. These residues from the manufacturing process are utilized as raw materials in a number of industries to create fertilizers, additives for improving the soil, animal food, paper, synthetic wood, and other products^29^. However, a sizable portion of the produced agricultural waste goes unused, causing residue to build up in the fields and preventing farmers from using the land. In this situation, farmers search for inexpensive, straightforward, and quick methods to get rid of the trash, such as burning them, this fills the air with a lot of smoke and CO_2_ emissions^30^. Crop residues have been utilized as a precursor for the manufacture of activated carbon, cement additive, and a reservoir for producing biofuels. Agro-waste, livestock wastage, and agro-industrial products all naturally rise as a result of the large increase in agricultural production that is required by rapid population expansion^31^. In the Asian and Pacific area, for instance, China produces the most agro-waste, or agricultural byproducts, at a rate of 842 million tons year, followed by India at a rate of 560 million tons annually. China generates 587 million tons of agricultural waste annually, with rice, corn, and wheat making up more than 80% of these leftovers^32^. The residue capacity of agro-waste is also greater in Pakistan, rice produces 19,714,000 million tone, cotton produces 39,632,000 million tone, wheat produces 45,384,000 million tone, sugarcane produces 21,018,00 million tone, maize produces 9,832,000 million tone, millet produces 798,000 million tone, barley produces 93,000 million tone, dry chilly produces 304 million tone, walnuts produces 5.2,000 million tone, pistachio produces 400 million tone, peanuts produces 26,400 million tone, coconuts produces 5100 million tone, castor oil seed produces 2200 million tone, peaches produces 27,200 million tone, papayas produces 1600 million tone, plums produces 16,400 million tone, rape seed produces 97,400 million tone, and sun flower seed produces 202,200 million tone^33^.

### Municipal solid waste

Municipal waste, also referred to as "rubbish" or "garbage," consists mainly of household waste and similar materials. It's composed predominantly of food waste, metals, fabrics, paper, glass, and plastic, which are produced by various entities including homes, educational institutions, medical facilities, hospitality establishments, businesses, and retail outlets^34^. This waste can be managed either by municipal authorities or by independent parties. Additionally, private sector entities like corporations or non-profit organizations may take the initiative to collect this waste for energy generation or material recovery, rather than relying solely on governmental entities^35^. However, municipal solid waste (MSW) does not encompass waste originating from municipal sewage systems and treatment plants, or waste generated from municipal construction and demolition activities^36^. The rates at which MSW is produced can vary with the season and city, and are generally reflective of the level of activity and economic prosperity^37^. In cities with higher income levels, per capita MSW production rates (kg/person/day) tend to be higher, with waste often containing bulky items such as furniture, abandoned vehicles, and packaging materials, in stark contrast to cities with lower income levels^38^.

### Industrial waste

Modern squanders contain a different range of materials with fluctuating levels of poisonousness to the climate. Bundling materials, paper, food handling waste, solvents, oils, paints, pitches, muck, metals, glass, stones, earthenware production, plastics, calfskin, elastic, wood, straw, texture, abrasives, and different materials fall into this class^39^. Exact output rates are unclear because to a lack of continuous, thorough, and up-to-date tracking of industrial garbage. Because raw materials, industrial processes, finished products, and environmental issues vary greatly between sectors, establishing common criteria that define industrial wastes in general is difficult^40^. Food processing, meat, chips, and juice are just a handful of the businesses that produce massive volumes of organic waste each year. As the world's population expands, so does the need for food products. Thus, a few drink and food organizations have extended decisively all over the planet to satisfy this need^41^. Subsequently, there are more squanders delivered every year because of their high content of lignin, hemicellulose, cellulose, nitrogen, carbon, ash, and moisture, fruit industrial wastes are increasingly being used as raw materials for the production of other valuable products, which can be biochemically digested to produce bio-ethanol, biogas, and other products^42^. Indeed, even among emerging nations, not simply across nations at various progressive phases, the age of modern garbage fluctuates^43^. For example, in China, the production ratio of MSW to industrial wastes is one to three; however, in other countries with comparable per capita wealth, the ratio is far lower. This amount of industrial rubbish is expected to increase in the next 20 years if current development rates continue^44^. Many nations' current industrial waste collection, processing, and disposal systems are inadequate however, this thing is viewed as a major source of worry^45^.

### Hazardous waste

Because of headways in various regions, like farming activities, modern plants, and medical care offices, the age of dangerous squanders is consistently expanding. Because of this turn of events, critical volumes of unsafe synthetic substances are consumed^46^. For instance, there are around 110,000 unmistakable perilous mixtures available today^47^. However, around a thousand novel compounds are launched each year for usage in a wide range of applications^48^. Chemicals, light bulbs, batteries, auto components, and discarded medications are examples of hazardous waste^49^. Clinics, thermal energy stations, and medical care offices all add to the development of unsafe poisons^50^. The most harmful toxins are produced by petrochemicals, chemicals, and petroleum facilities. In addition, significant contributors to this waste category include power generation facilities, pulp and paper factories, metal fabrication and milling centers, as well as wood processing plants. Leather manufacturing processes are notably recognized for releasing harmful pollutants like chromium ions into wastewater streams, given their extensive production volume^51^. The production and application of pesticides also considerably contribute to the generation of hazardous waste^52^. In the Asian and Pacific region, the notable hazardous wastes are waste solvents, wastes rich in chlorine, pesticides like organophosphates, and wastes that contain significant levels of solvents, chlorine, and pesticides^53^. The National Hazardous Waste Management Policy, 2022 of Pakistan is a set of guidelines for the environmentally sound management of solid and hazardous waste in the country. The Ministry of Climate Change has formulated this comprehensive national level policy through consultative process with relevant stakeholders. This Policy is aimed at acting as an umbrella document to address the issue of hazardous waste. The decision has been taken as Pakistan annually produces 30 million tonnes of waste in addition to annually importing 80,000 tonnage of bundled waste from around the world, which has been causing environmental and health problems as well as contaminating the surface water and groundwater supplies^54^.

## Solid waste production, management and utilization

### Solid waste production

In an extensive study covering 367 countries, the World Bank conducted an in-depth analysis of waste generation and its management around the world^55^. Table 1 outlines the total volume of solid waste generated in each region in 2016 and how it was categorized^56–58^. Approximately 2 billion tons of solid waste was produced in that year, and projections suggest this figure could surge to 3.40 billion tons by 2050 due to increasing population and urbanization trends^59^. The data suggests that the East Asia and Pacific region generated the highest annual volume of waste, totaling 468 million tons, equating to an average daily per capita generation of 0.56 kg^60^. Europe and Central Asia followed, producing 392 million tons of waste annually, or 1.18 kg per person per day. The majority of the waste consists of organics and solid recyclables such as paper and plastics^61^. The region producing the least amount of waste is the Middle East and North Africa, contributing only 6% to the global waste production with 129 million tons annually^62^. With financial turn of events and populace development, squander creation is probably going to increase. Low-pay nations are expected to see the most development^63^. In the next 30 years, trash levels are anticipated to twofold in Sub-Saharan Africa and fourfold in South Asia^64^. The quantity of waste generated in higher-income nations is expected to decrease. Future trash generation projections are worrying and depressing, since growing garbage amounts will place further burden on the ecosystem, necessitating immediate actions to minimize waste amounts and encourage the usage of various waste management methods^65^.

**Table 1:** Solid waste classification and capacity around the world in 2016^56–58^.

Population and waste type	Population (million person)	Plastic (%)	Paper and cardboard (%)	Food (%)	Rubber and leather (%)	Glass (%)	Metal (%)	Wood (%)	Others (%)
Middle East and North Africa	0.44	12	13	58	2	3	3	1	8
Sub-Saharan Africa	1.04	9	10	43	0	3	5	1	30
Latin America and Carribbean	0.64	12	13	52	0	4	3	1	15
North America	0.37	12	28	28	9	5	9	6	4
South Asia	1.76	8	10	57	2	4	3	1	15
Europe and Central Asia	0.91	12	19	36	1	8	3	2	21
East Asia and Pacific	2.29	12	15	53	1	3	3	2	12

### Solid waste management

The waste management sector, encompassing municipal, industrial, and hazardous waste, has shown robust growth, with a market value of 2080 billion dollars in 2019, projected to increase to 2339.8 billion dollars by 2027^66^. This field involves the collection, processing, and disposal of waste. The initial step in waste management is the collection of waste, which demonstrates the degree of effort invested in the process. This service, which can be public or private, operates in various forms. One such method is door-to-door collection, where trucks or waste collection vehicles collect waste directly from residences and markets^67^. In other situations, waste is collected at a central point and subsequently transported for further management^68^. According to data from the World Bank, almost 100% of waste is collected in high-income cities. However, this figure decreases for cities with lower income levels: upper-middle-income cities show 82% collection rates, lower-middle-income cities report 51%, and low-income cities have rates around 39%^69^. Squander assortment rates in metropolitan regions were additionally observed to be more prominent than in provincial ones. A few metropolitan regions have rates that are over two times as high as rustic regions in a similar city^70^. Uncollected garbage is ordinarily discarded by open unloading, which has adverse ramifications for the climate and human wellbeing^71^. The wastes collected are treated using the different methods including landfilling, biological treatment (compositing and anaerobic digestion) and physic-chemical method (combustion, sterilization, pyrolysis, and gasification). Globally, about 40% of waste finds its way to landfills, while 19% is recycled or composted. Modern incineration accounts for the processing of 11% of waste, leaving 37% that ends up in dumps^72^. The income level of a region significantly influences waste collection and disposal methods. For instance, in countries with lower income levels, where proper landfill sites are not available, open spaces and streets often become default dumping grounds, with over 70% of waste treated in this manner. Regions like Sub-Saharan Africa and South Asia account for more than 66% of all improperly discarded waste globally^73^. Conversely, in high and upper-middle-income countries, better waste treatment strategies such as regulated landfill use and recycling are more common. Upper-middle-income countries have the highest reliance on landfills, averaging 54%. High-income countries, on the other hand, depend less on landfills, disposing of 39% of their waste this way, thanks to the adoption of more economically viable methods such as recycling and composting (25% of waste) and incineration (22% of waste)^74^. Challenges to effective waste management include inadequate planning and evaluation, lack of government coordination, harsh work environments, and space constraints given the extensive land area required for processes like landfilling^75^.

### Solid waste utilization

The purpose of this article is to discuss the usage of solid waste to generate electricity. To that goal, a number of solid waste power generating systems were assessed using techno-economic criteria as compared to the other methods stated in Table 2. The gasification strategy for power age is appropriate. The gasification technique takes a wide range of junk for power age and delivers less debris. It likewise has a more powerful efficiency^76^. Gasification, with its hybrid system, opens the door to yet another new development in the country, as hybrid technologies based on other resources such as coal, combined with biomass and solid waste resources, provide community with energy advantages^77^.

**Table 2:** Technology selection parameters for power generation.

Technology options	Efficiency (%)	Unit capacity (KWh/tone)	Disposal	Waste type	Output	Operating cost per tone	Capital cost per tone
Incineration^78^	25	850	5% bottom ash	Process homogeneous waste	Energy and heat	$ 60	$ 775
Pyrolysis^37^	18	800	0.3% bottom ash	Process homogeneous waste	Energy and syngas	$ 150	$ 1500
Gasification^79^	30	800	1% bottom ash	Process heterogeneous waste	Energy and syngas	$ 60	$ 850
Plasma^80^	10	600	10% bottom ash	Process homogeneous waste	Energy and syngas	$ 120	$ 1300

## Materials and methods

### Research area

With a population of 207 million and a 2.4% annual growth rate, Pakistan is a country in the northwest of South Asia, Its territory is 881,913 km^2^. China shares boundaries with the nation's northeast, India with it on the east, Iran and Afghanistan on the west, and the Arabian Gulf with it on the south^81^. The real Gross Domestic Product (GDP) of Pakistan is increasing at a rate of 5.8% annually. By 2050, the nation will rank fourth in the globe in terms of population, if population growth continues at its current pace of 2.4% per year^82^. Approximately 500 kWh of electricity is consumed per person annually, which is extremely low when compared to the global average of 2603 kWh. In 2007, it was estimated that there would be between 1 and 2 GW of an electrical deficit, but by 2022, there were 4 GW of shortages^83^.

The majority of Pakistan’s energy output depends on fossil fuels. Hydropower, other renewable energy source (solid waste, biomass, wind and solar), and indigenous coal all have a bright future in the country, but they have not been used to their full potential because of various technical, economic, and political roadblocks^84^. The country’s installed power generation capacity has only increased from 19,420 MW in 2008 to 34,605 MW in 2020^85^. Pakistan should put its attention on managing the potential of solid waste and other renewable energy resources if it wants to increase the amount of sustainable energy sources in its mix for generating electricity. Pakistan is one of the ten countries affected by climate change the most. In order to address both climate change and the world’s rapidly expanding energy demand, an appropriate energy mix is required^86^.

### Research method

**Figure 1:**
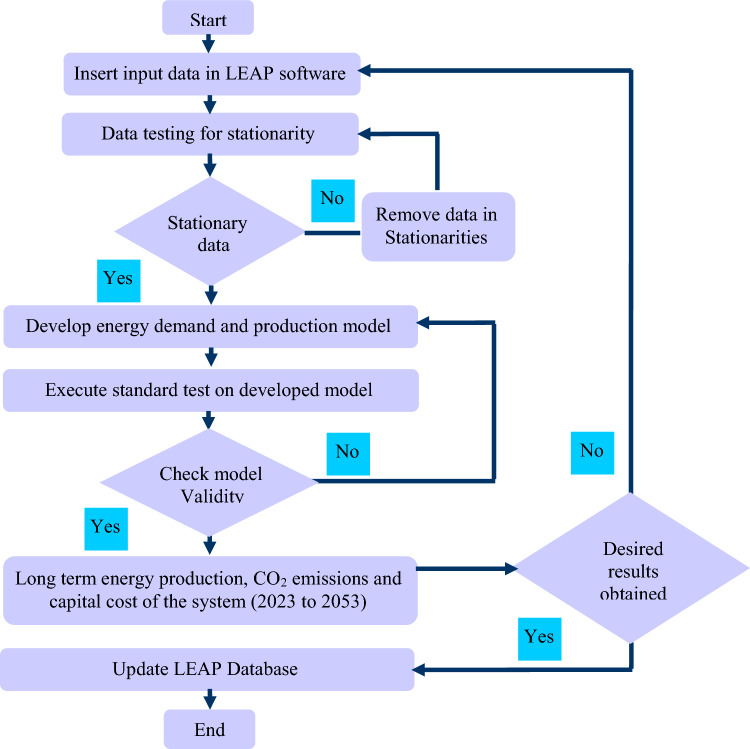
Methodological flow diagram.

Accordingly, this study develops energy transition pathways for Pakistan between 2023 and 2053 that use solid waste as a fuel source. Figure 1 displays a flowchart for an operation.

### Research data and forecast preparation

**Figure 2:**
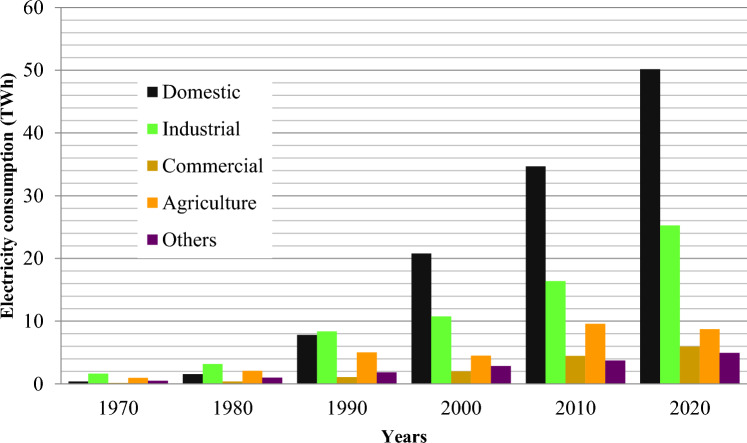
Past electricity consumption data from 1970 to 2020 in terawatt hours^88^.

Aspects in the social, technical, and demographic spheres affect CO_2_ emissions, capital cost and production^87^. Table 3 demonstrates important input variables for the LEAP energy generation module however, the past consumption of electricity (1970–2020) is given in Fig. 2^88^. The LEAP module includes exogenous characteristics for the lifespan of energy technologies, the development of electricity consumers, fuel prices, and GDP growth. In the LEAP module, endogenous features include sectorial energy demand, solid waste generation capacity, and electricity intensity^89^. New versions of LEAP (2020.1.32) are used in this research^88^.

**Table 3:** Key input parameters for energy production module in LEAP (2022)^88^.

Power plants	Capacity (GW)	Generation (GWh)	Efficiency (%)	Maximum availability (%)	Life time	CO_2_ emissions per fuel type (kg/Gj)
Solid waste	1.467	564.46	35	80	30–35	–

**Figure 3:**
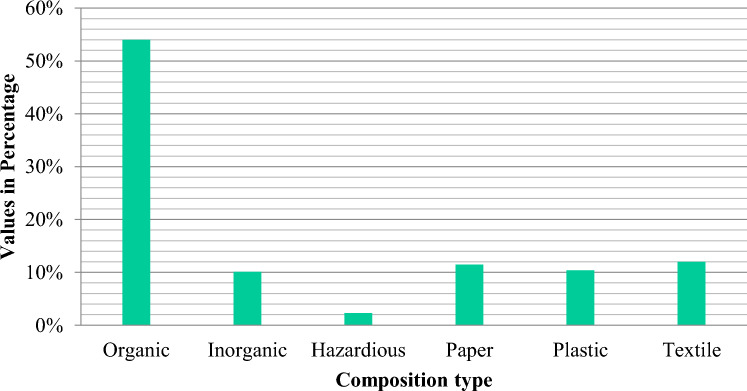
Composition type of solid waste in Pakistan^73^.

**Figure 4:**
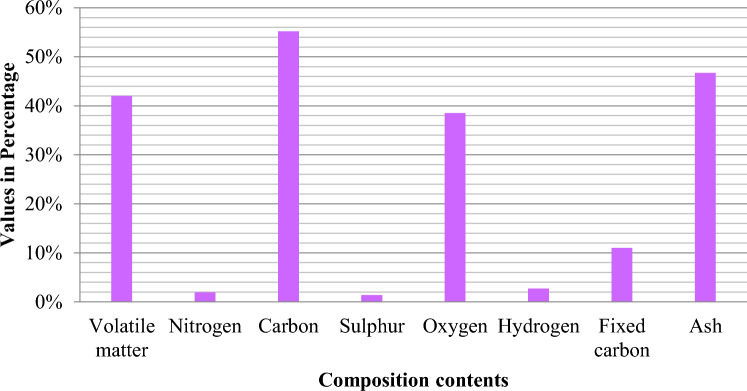
Composition contents of solid waste in Pakistan^90^.

Figure 3 indicates the kind of waste content and its percentage. To determine the chemical composition of solid waste products, physiochemical characteristics are used. The selectivity and acceptability of solid waste as a fuel source could be determined using these parameters^73^. Figure 4 shows how solid waste is physiochemically. To ascertain the physicochemical properties, proximate analysis test measures fixed carbon, ash, moisture, and volatile matter in total solid waste and the ultimate analysis test measures the proportion of oxygen, nitrogen, sulphur, carbon, and hydrogen in total solid waste^90^.

### Experimental setup

**Figure 5:**
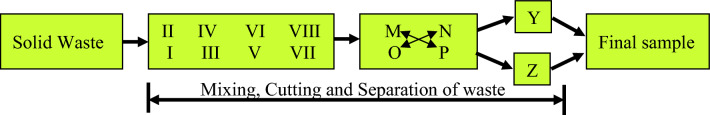
Quartering method diagram for waste sampling.

As demonstrated in the construction of an experimental environment using the quartering method. Figure 5 shows 50 kg of solid waste were initially collected from various locations throughout Pakistan. The material was then collected in one location after physical mixing and cutting. The entire waste were divided into eight groups, labelled I, II, III, IV, V, VI, VII, and VIII. Additionally, these eight sections were split between sections that were even (II, IV, VI, and VIII) and odd (I, III, V, and VII). Similar to this, odd pieces were grouped together and assigned to the O and P groups and also, even pieces were grouped together and assigned to the M and N groups. The four pieces M, N, O, and P were then diagonally mixed to create M & P and N & O, respectively, before being separated into two groups, M & P into Y and N & O into Z. Finally, Y and Z were combined to create the final analytic sample. Up until the weight reached 30 kg, manual mixing and cutting were done multiple times.

### Theoretical and mathematical framework for power potential from solid waste based resource

The calorific value is measured in energy per unit volume and is highly dependent on the amount of heat produced during the combustion process. The calorific value of the final sample of waste pellets (30 kilo gram) was determined in the laboratory using the Gallen Kamp Ballistic Bomb (GKBB) Calorimeter. The net value of calorific is determined as 6519 Kilocalorie/kg. However, for other research data, the range of calorific value is 9 MJ/Kg to 44 MJ/Kg respectively. Further Eqs. (1) and (2) was used for finding the higher and lower values of calorific of waste pallets^91^.

1 $$\mathrm{Higher \;calorific \;value }= \frac{\sum \mathrm{Qp }\left({\text{C}}.{\text{V}}\right){\text{H}}}{{\text{Tp}}}$$

2 $$\mathrm{Lower \;calorific \;value}= \frac{\sum \mathrm{Qp }\left({\text{C}}.{\text{V}}\right){\text{L}}}{{\text{Tp}}}$$

where; (C.V)L = Lower calorific value in kilocalorie/kg, (C.V)H = Higher calorific value in kilocalorie/kg, Qp = Quantity of specific material in the total waste pallets in kg and Tp = Total waste pallets in kg. The power potential of mixed solid waste pallets can be calculated by Eq. (3)^92^.

3 $${\text{Ep}}=\left({\text{C}}.{\text{V}}\right)\mathrm{L }\times \mathrm{Aw }\times 1.16$$

where; Ep = Energy potential in kWh and Aw = Aggregate waste in kg.

## Results and discussion

**Figure 6:**
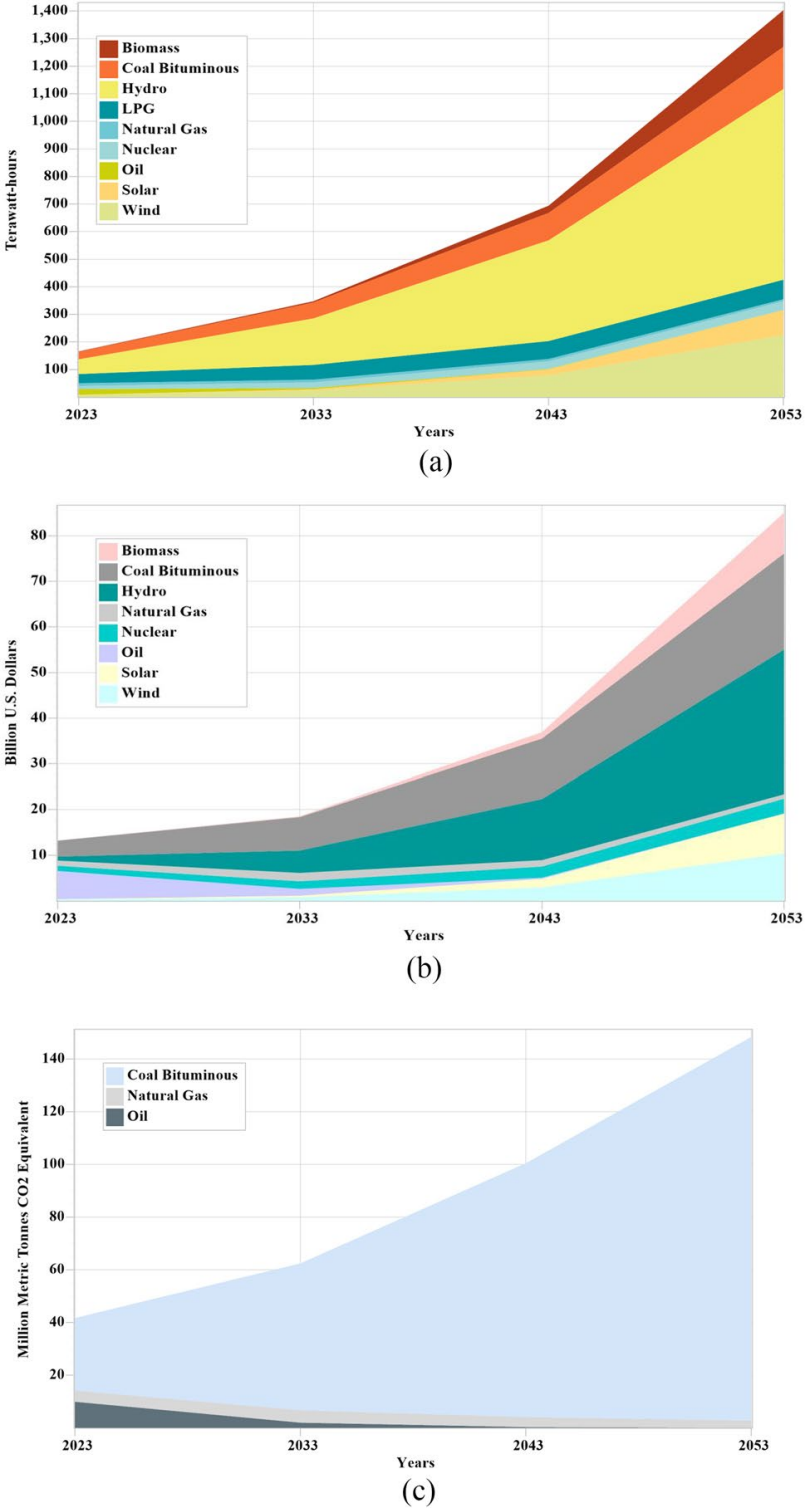
(**a**–**c**) Electricity production, capital cost and CO_2_ emissions under the 20% growth rate of solid waste based electricity.

**Figure 7:**
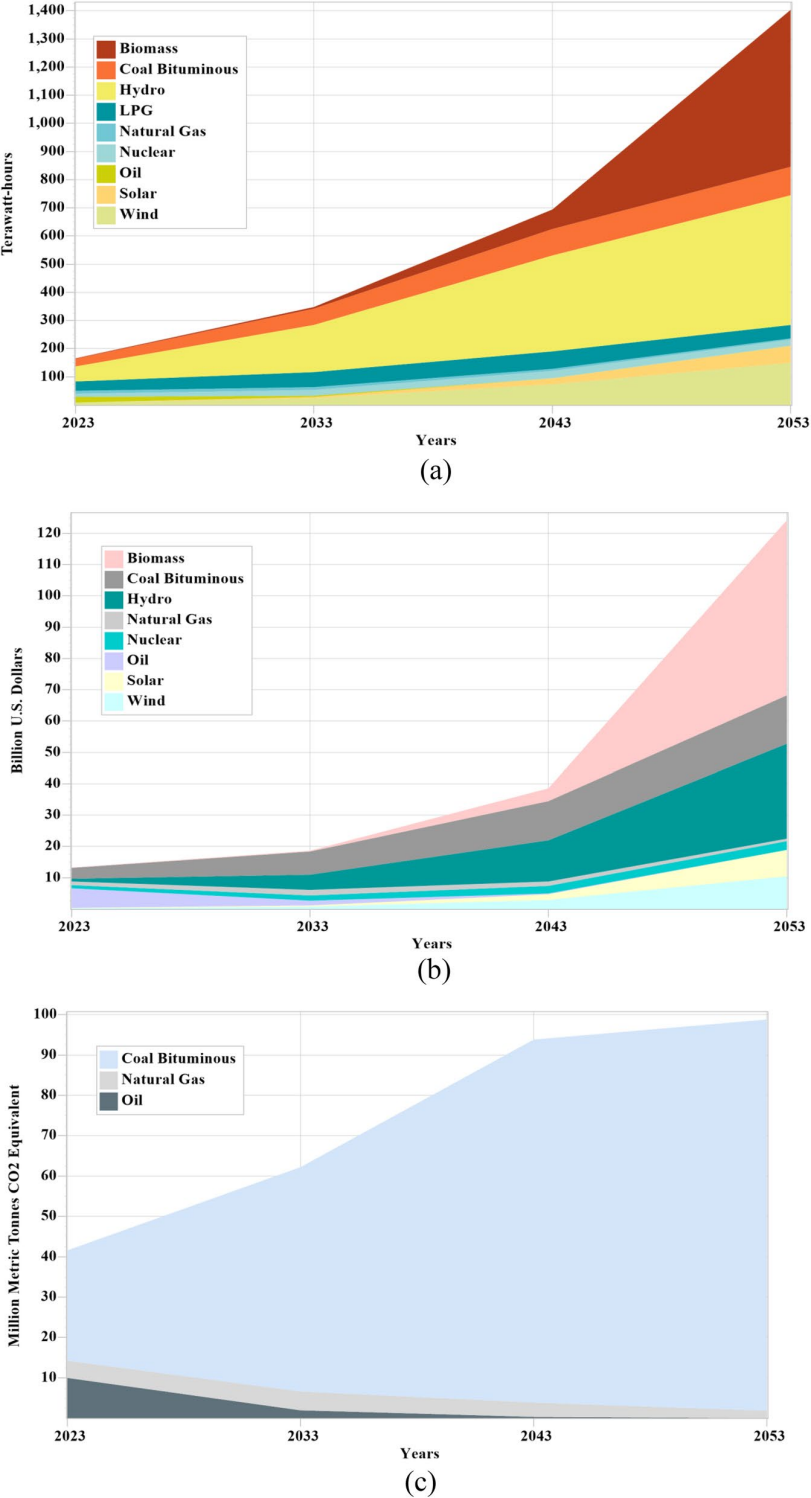
(**a**–**c**) Electricity production, capital cost and CO_2_ emissions under the 30% growth rate of solid waste based electricity.

**Figure 8:**
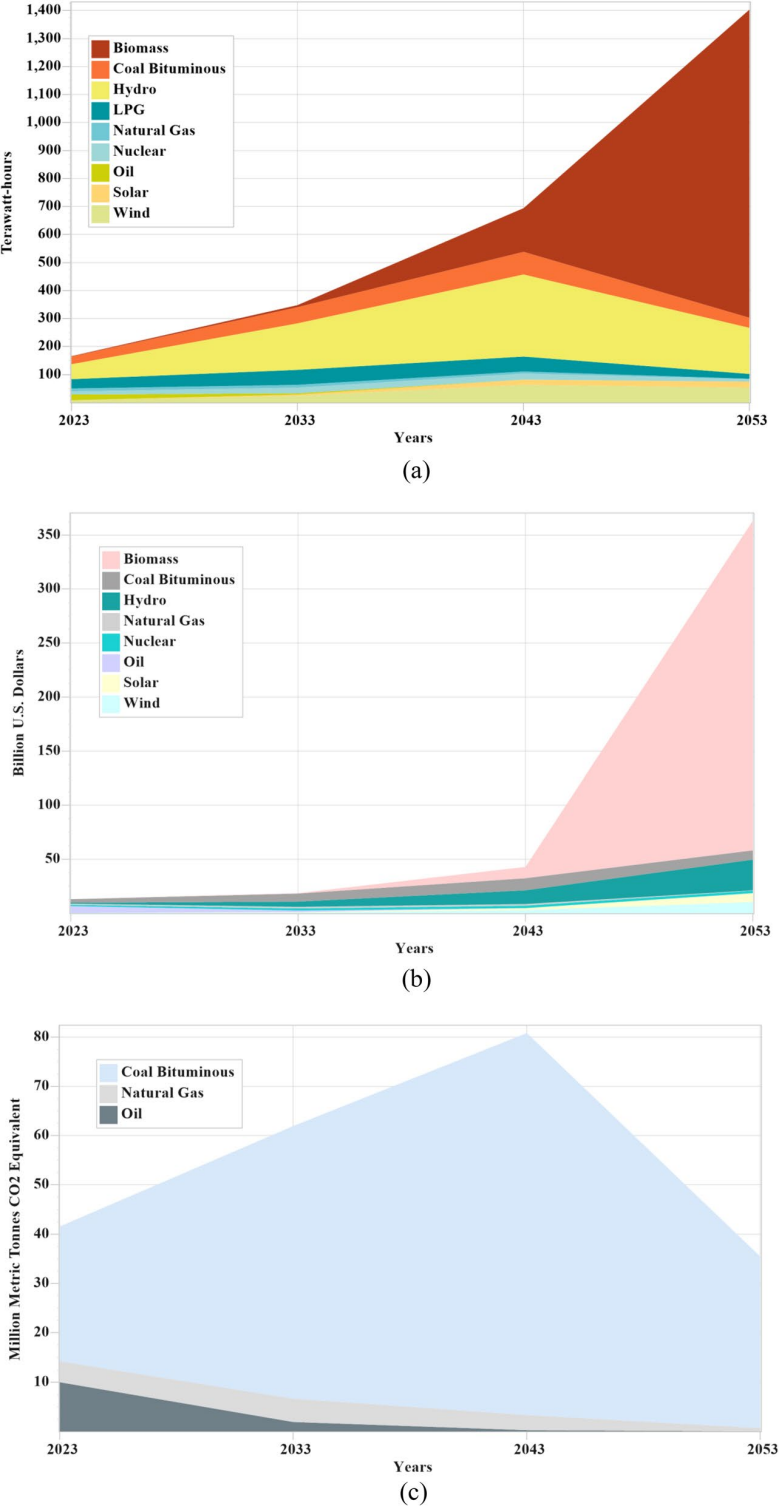
(**a**–**c**) Electricity production, capital cost and CO_2_ emissions under the 40% growth rate of solid waste based electricity.

Forecasts for electricity output, capital costs, and CO_2_ emissions are presented in Figs. 6a–c, 7a–c, and 8a–c for the years 2023–2053. The total gross electricity generated less the electricity used for auxiliary systems, self-consumption and the losses in transmission and distribution systems is the total net electricity demand. As a result, the projection for the whole net demand reveals notable variations. The predicted energy demand takes a deceleration in consumption of 955.14 TWh till 2053 into consideration. However, the nation's total projected power output of 1402.97 TWh will be sufficient to cover all of the country's energy needs through the year 2053. The residential sector has the highest electrical demand, followed by the industrial, commercial, and public service sectors. Pakistan’s electrical generation rapidly evolves to enable the energy system transition, moving from a 62.1% predominance of fossil fuels in 2023 to 81.3% renewables in 2053 and completely zero CO_2_ emissions in 2060. The cost of electricity producing technologies is the motivating factor. Solid waste becomes a major source of electricity in a cost-effective energy transition, rising from 0.81% in 2023 to 9.44% by 2053 under the 20% growth rate, then to 39.67% under the 30% growth rate, and finally to 78.33% under the 40% growth rate, as shown in Figs. 6a, 7a, and 8a. The outstanding resource distribution in Pakistan's rural and urban areas is also responsible for the exponential development in the supply of solid waste-based electricity. Hydropower is the main renewable energy source in the early stages of the transition, with a share in electricity supply rising to 49.31% under projects based on solid waste growth of 20%, declining to 32.85% under projects based on solid waste growth of 30%, and finally becoming 11.79% under projects based on solid waste growth of 40% until 2053. After that, wind and solar power become more economical. By 2053, wind and solar will have some responsibilities in Pakistan's electricity mix, playing complimentary functions as the country transitions to a more renewable energy source. They will also play significant roles in the country's energy supply. The contribution of wind and solar energy increases to 16.05% and 6.48% under the 20% share of solid waste based projects until 2053 before gradually declining to about 10.96% and 4.32% under the 30% share of solid waste based projects and finally to 3.84% and 1.55% under the 40% share of solid waste based projects.

Under the 20% share of solid waste-based projects, capital cost of solid waste-based projects are increases significantly on an annual basis from 0.04 b$ by 2023 to 0.19 b$ by 2033, 1.41 b$ by 2043 and 8.88 b$ by 2053 as shown in Fig. 6b. However the total capital cost is also increasing from over 13.23 b$ in 2023 to 18.59 b$ by 2033, 36.96 b$ by 2043 and 85.01 b$ by 2053. Under this condition, the total CO_2_ emissions increase from over 41.60 million metric tons to 62.44 million metric tons by 2033, 100.34 million metric tons by 2043, and 148.26 million metric tons by 2053 as shown in Fig. 6c. Under the 30% share of solid waste-based projects, capital cost of solid waste-based projects are increases significantly on an annual basis from 0.04 b$ by 2023 to 0.25 b$ by 2033, 4.05 b$ by 2043 and 55.90 b$ by 2053 as shown in Fig. 7b. However the total capital cost is also increasing from over 13.23 b$ in 2023 to 18.61 b$ by 2033, 38.58 b$ by 2043 and 124.14 b$ by 2053. Under this condition, the total CO_2_ emissions increase from over 41.60 million metric tons to 62.22 million metric tons by 2033, 93.85 million metric tons by 2043, and 98.76 million metric tons by 2053 as shown in Fig. 7c. 33% less CO_2_ emissions are produced under this condition as the capacity of renewable sources increases. Under the 40% share of solid waste-based projects, capital cost of solid waste-based projects are increases significantly on an annual basis from 0.04 b$ by 2023 to 0.33 b$ by 2033, 10.66 b$ by 2043 and 304.98 b$ by 2053 as shown in Fig. 8b. However the total capital cost is also increasing from over 13.23 b$ in 2023 to 18.64 b$ by 2033, 43.09 b$ by 2043 and 363.11 b$ by 2053. Under this condition, the total CO_2_ emissions increase from over 41.60 million metric tons to 61.96 million metric tons by 2033, 80.76 million metric tons by 2043, and further it reduces 35.46 million metric tons by 2053 as shown in Fig. 8c. 62% less CO_2_ emissions are produced under this condition as the capacity of renewable sources increases.

We may build energy policies that serve as the foundation of sustainable energy governance based on the results of the energy variable simulations under the assumptions of a 20%, 30%, and 40% expansion of solid waste-based electricity projects initiatives from 2023 to 2053 in Pakistan. These regulations are designed to lower emissions, improve energy efficiency, and support a steady transition to renewable energy. Understanding the effects of renewable energy sources and energy policies on Pakistan’s energy governance is made possible by this analysis. With tight integration between diverse hydrocarbon industry components like exploration, extraction, transportation, burning, and retailing, the modern fossil fuel economy demonstrates the characteristics of a mature socio-technical system. None of the present models of energy governance can neglect environmental stewardship, and the energy industry continues to face multiple issues, according to an assessment of the key international accords, regulations, and publications on energy governance. The understanding that politics and political processes are essential to governance for sustainable development is a recurring subject in these debates. Global energy policy is urgently needed to address climate change, geopolitical tensions, and economic fragility since it is affecting every continent and nation and causing disruptions to national economies and lives. A radical revamp of the energy system is necessary to realize a sustainable energy future.

Energy is a foundational element in all dimensions of development, yet sustainable energy is critical for enhancing the well-being and living conditions of millions globally. Hence, there is an immediate requirement for advancements in access to modern sustainable energy to facilitate nations' progress. Formulating energy policies in line with clean energy consumption, enhanced energy efficiency, and responsible usage is crucial in shaping sustainable energy governance. Being a country that imports fossil fuels, Pakistan's energy structure has displayed a high dependency on fossil fuels and their derivatives, accounting for a significant portion of the country's total expenditure. In recent times, a significant shift in its energy approach has been observed, possibly due to projections indicating a declining phase for fossil fuel resources, with estimates suggesting that reserves could sustain for about 5 to 10 more years^93^. The country's emphasis on renewable energy development has centered on solid waste based energy, with investments channeled into multiple related projects. Pakistan's potential for bioelectricity generation has been estimated to be around 20 GW.

The government faces a huge task in making the switch from a strong reliance on fossil fuels to self-sufficiency through renewable energy. Policies that encourage innovation in sustainable energy alternatives like solar, wind, and biofuels are necessary. Establishing rules that permit infrastructure projects that ensure an energy supply while upholding good standards is essential. The use of renewable energy has the potential to significantly reduce the production and use of energy sources with a high carbon footprint. Mitigating climate change requires a quick transition to a low-carbon economy based on renewable energy. Localized reductions in air pollution and accompanying harmful health consequences may occur immediately as a result of climate-oriented measures that reduce energy-related CO_2_ emissions. By encouraging the use of renewable energy, CO_2_ emissions could be reduced by discouraging the use of fossil fuels. Due to the long-term positive equilibrium link between the use of renewable energy sources and economic growth, reducing CO_2_ emissions is essential to achieving sustainable economic growth. Many studies as shown in Table 4 have revealed a strong correlation between the use of renewable energy, reduction in CO_2_ emissions and a country’s economic development through energy transition. Promoting policies related to energy transition could promote nations’ sustainable economic growth.

**Table 4:** Energy transition studies conducted in various countries.

Country and references	Study focus
New york, USA^94^	100% renewable electricity transition planning is conducted using a data-driven multistage adaptive robust optimization approach with machine-learning. The biomass/solid waste was not considered but the relationship of wind, solar, and hydropower with CO_2_ emissions is presented
USA^95^	This study evaluated the relationship of renewable generation, energy storage and energy efficient technologies to enable carbon neutral energy transition using wind, solar, biogas, hydropower, biomass, and geothermal potential
China^96^	This study suggested that China's energy transition strategy at the city level is possible by incorporating the hydropower, wind, solar, geothermal, and biomass potential
Iran^97^	This study Investigated the public acceptance and willingness to use renewable energy sources through socio-psychological model for reducing CO_2_ emissions by exploiting hydropower, wind, solar, geothermal, and biomass sources
India^98^	Transition towards Renewable Energy Production is recommended in this study by exploiting the potential of solar, biogas, biomass, hydropower, and geothermal sources
Germany^99^	Reconciling renewable energies (solar, wind, hydropower) with human wellbeing and nature in the German Energy Transition
Mexico^100^	A transition strategy from fossil fuels to renewable energy sources (wind, solar, biogas, biomass, hydropower, geothermal) in the Mexican electricity system is suggested in this study
Japan^101^	National and local imaginaries in Japan and Fukushima around transitions to hydrogen fuel and renewables are suggested in this study
Europe^102^	The role of the agriculture sector in renewable energy transitions is promoted in this study
Norway^103^	Transitioning to renewable energy systems through the use of solar, wind, hydrogen energy is recommended in this study
Our proposed study, Pakistan	Our study is focused on the role of solid waste in the transition to low carbon future in Pakistan. The utilization of solid waste as a feedstock, such as agricultural waste, municipal solid waste, industrial waste, and hazardous waste was examined. Building power plants based on solid waste resource is essential in rural and urban sites of Pakistan for the alleviation of energy crises. This study set out to explore Pakistan's potential for producing renewable energy from solid waste and estimating the future carbon emissions and capital investment cost from 2023 to 2050 using the LEAP energy modelling tool. Three estimates have been considered in forecasting like 20%, 30% and 40% share of solid waste based electricity for sustainable energy mix and forecasted the future carbon emissions and total capital investment cost accordingly

Many growing economies are significantly dependent on fossil fuels for their production systems. Under such frameworks, the transition to cleaner energy sources is often insufficient. Thus, increasing the usage of clean energy in manufacturing processes can help lessen the negative environmental impact of economic growth. Regulatory interventions should be implemented to foster clean energy as a viable alternative to conventional energy sectors. Such measures can result in a variety of positive outcomes, such as more employment opportunities, higher energy security, improved economic growth, and the emergence of export focused enterprises, all of which have a positive impact on the environment. Many homes in underdeveloped nations still utilise solid fuels like firewood and dung cake for cooking, even in urban areas where it is assumed that other energy sources are available. Since cleaner biomass solution are frequently more expensive, government help is essential for household adoption. In less developed nations, clean energy is a crucial source of energy, but there is room for improvement in terms of efficiency, cost and CO_2_ emissions reductions. There is significant potential to reduce CO_2_ emissions through a variety of bioenergy alternatives, including municipal solid waste, bioenergy crops, agricultural byproducts, and rice husks.

The amount of fossil fuel utilised and how effectively energy is produced from solid waste depend on the possible decrease in CO_2_ emissions. Therefore, government agencies should support programmes that aim to improve feedstock availability and conversion efficiency through solid waste innovation. However, shifting energy policies away from reliance on fossil fuels and towards acceptance of renewable energy sources is necessary to reduce CO_2_ emissions. Due to an increase in revenue expenditure, this change may result in a drop in surplus revenue. Levying a fee on business that produce pollution and rely on fossil fuels as their source of energy could serve as a potential counter balance to this. These polluting industries may explore switching to cleaner, alternative energy sources as the carbon tax reduces their appeal.

It's essential to bear in mind that while we strive to reduce CO_2_ emissions via greater adoption of cleaner production methods, we must not compromise on environmental integrity. Clean energy often originates from biological materials sourced from various places, including wood and agricultural products. Over exploitation of these resources could lead to land degradation and deforestation, either directly or indirectly. Policies that encourage clean energy development are typically linked with objectives like mitigating climate change, enhancing energy access, boosting energy security, and promoting economic growth. However, as the clean energy sector expands, several obstacles remain. For instance, the use of edible crops for biofuel production raises significant concerns about potential effects on food security. Moreover, cleaner energy production can have negative environmental impacts, affecting water quality and availability, CO_2_ emissions, soil erosion, and biodiversity. The Government of Pakistan announces the Alternative Renewable Energy (ARE) 2019 policy, as a component of the overall plan, has a vision of the development of an efficient, sustainable, secure, affordable, competitive and environment friendly power system while promoting indigenization of energy resources and development of local manufacturing capabilities in such technologies. Pakistan's ARE 2019 sets several overall objectives and some specific targets for the energy sector in the country. The first objective of increasing the share of renewable energy is accompanied by a target of increasing renewable energy generation to 20% by 2025, and then 30% by 2030^104^.

Policymakers can consider numerous strategies when formulating regional or national clean energy policies, and the insights gained from this study suggest several potential avenues for future research. The focus of upcoming energy policies is anticipated to encourage the shift from fossil fuels towards renewable alternatives, such as solid waste. However, in light of the recent COVID-19 crisis, there are worries that some economies might roll back fuel standards or green stimulus funds, which could lead to a reduction in solid waste utilization and its subsequent growth. While it is our hope that this scenario will not transpire, if it does, the proportion of solid waste use could gradually decline. As such, an interesting area for future research would be to investigate the impact of COVID-19 on the usage of solid waste. Lastly, while the findings of this study are pertinent to one specific type of renewable energy source, namely solid waste, it would be beneficial to extend this research to encompass other renewable energy sources such as solar, wind, and hydropower.

## Conclusion and policy implications

Utilising LEAP software for the study period of 2023–2053, the current study evaluated the solid waste power potential under the 20%, 30% and 40% growth rates of solid waste projects in the total energy mix of Pakistan. Using time series dependency analysis, LEAP software has capability to provide country specific information regarding the relationship between energy usage and CO_2_ emissions. The following is a list of the study’s principal conclusions.

- In the beginning, the annual increase in power consumption was over 8% yearly, with a 1193.93 TWh demand predicted for 2053. However, the nation's total projected power output of 1402.97 TWh will be sufficient to cover all of the country's energy needs through the year 2053.
- Pakistan electricity generation rapidly evolves to enable the energy system transition, moving from largely using fossil fuels (62.1%) in 2023 to 81. 3% renewables in 2053, and eventually to zero CO_2_ emissions by 2060.
- In a cost optimal energy transition, solid waste replaces hydro source, which are expensive and seasonal, as the main source of electricity. It rises from a mere 0.81% in 2023 to about 9.44% by 2053 under the 20% growth rate, then rises to 39.67% by 2053 under the 30% growth rate, and finally rises to 78.33% by 2053 under the 40% growth rate.
- Pakistan generates 0.8% of the world’s carbon footprint, but in 2023, we are among the ten most climate stressed nation. The total CO_2_ emissions from 2023 to 2053 are reduced thanks to this analysis to 35.46 million metric tones from 148.26 million metric tones, while the capital cost rises from 13.23 billion dollars in 2023 to 363.11 billion dollars in 2053.
- This paper presents an energy transition pathway that might take Pakistan from its existing fossil fuel based energy system to one that is economical, effective, sustainable and secure.

This study offers numerous advantages for reducing environmental impact and promoting sustainable practices by implementing waste-to-energy system. It contributes to renewable energy generation, waste diversion and reduction, CO_2_ emission reduction, resource recovery, and economic benefits. In addition, the land used for landfill purposes could be utilized for many other useful purposes.

## Data Availability

Data will be made available on request to Corresponding Author.

## References

[1] Jamil MH (2022). Did the restructuring of the electricity generation sector increase social welfare in Pakistan?. Renew. Sustain. Energy Rev..

[2] Grainger CA, Zhang F (2019). Electricity shortages and manufacturing productivity in Pakistan. Energy Policy.

[3] Raza MA (2022). Holistic and scientific approach to the development of sustainable energy policy framework for energy security in Pakistan. Energy Rep..

[4] Abdullah FB (2022). An empirical analysis of sustainable energy security for energy policy recommendations. Sustainability.

[5] Abbasi KR, Abbas J, Tufail M (2021). Revisiting electricity consumption, price, and real GDP: A modified sectoral level analysis from Pakistan. Energy Policy.

[6] Shah SAA, Solangi YA (2019). A sustainable solution for electricity crisis in Pakistan: Opportunities, barriers, and policy implications for 100% renewable energy. Environ. Sci. Pollut. Res..

[7] Tursunov O (2020). Characterization of tar generated from the mixture of municipal solid waste and coal pyrolysis at 800 °C. Energy Rep..

[8] Raza MA (2022). Energy demand and production forecasting in Pakistan. Energy Strategy Rev..

[9] Saqib NU, Sarmah AK, Baroutian S (2019). Effect of temperature on the fuel properties of food waste and coal blend treated under co-hydrothermal carbonization. Waste Manag..

[10] Khatri KL (2021). Investigation of possible solid waste power potential for distributed generation development to overcome the power crises of Karachi city. Renew. Sustain. Energy Rev..

[11] Khan AH (2022). Current solid waste management strategies and energy recovery in developing countries-State of art review. Chemosphere.

[12] Yang Y (2021). Gasification of refuse-derived fuel from municipal solid waste for energy production: A review. Environ. Chem. Lett..

[13] Maghmoumi A, Marashi F, Houshfar E (2020). Environmental and economic assessment of sustainable municipal solid waste management strategies in Iran. Sustain. Cities Soc..

[14] Rehan M (2023). Untapping the potential of bioenergy for achieving sustainable energy future in Pakistan. Energy.

[15] Safar KM (2021). Integrated model of municipal solid waste management for energy recovery in Pakistan. Energy.

[16] Rehmani M (2020). Tariff determination for municipal waste management power projects in Pakistan. Waste Manag. Res..

[17] Jabeen F (2022). Trash to energy: A measure for the energy potential of combustible content of domestic solid waste generated from an industrialized city of Pakistan. J. Taiwan Inst. Chem. Eng..

[18] Rehan, M., *et al*. A sustainable use of biomass for electrical energy harvesting using distributed generation systems. *Energy*. 128036 (2023).

[19] Aslam S (2022). Application of material flow analysis for the assessment of current municipal solid waste management in Karachi, Pakistan. Waste Manag. Res..

[20] Korai MS (2020). Comparison of MSW management practices in Pakistan and China. J. Mater. Cycles Waste Manag..

[21] Mukherjee C (2020). A review on municipal solid waste-to-energy trends in the USA. Renew. Sustain. Energy Rev..

[22] Raza MA (2021). Harnessing electrical power from hybrid biomass-solid waste energy resources for microgrids in underdeveloped and developing countries. Eng. Technol. Appl. Sci. Res..

[23] Iqbal A (2023). Evolution of solid waste management system in Lahore: A step towards sustainability of the sector in Pakistan. Appl. Sci..

[24] Zhang H (2023). Hybrid deep learning model for accurate classification of solid waste in the society. Urban Clim..

[25] Tripathi N (2019). Biomass waste utilisation in low-carbon products: Harnessing a major potential resource. NPJ Clim. Atmos. Sci..

[26] Nanda S, Berruti F (2021). A technical review of bioenergy and resource recovery from municipal solid waste. J. Hazardous Mater..

[27] Kapoor R (2020). Valorization of agricultural waste for biogas based circular economy in India: A research outlook. Bioresource Technol..

[28] Kumar Sarangi P (2023). Utilization of agricultural waste biomass and recycling toward circular bioeconomy. Environ. Sci. Pollut. Res..

[29] Maji, S., *et al*. Agricultural waste: Its impact on environment and management approaches. *Emerg. Eco-friendly Green Technol. Wastewater Treatment*. 329–351 (2020).

[30] Duque-Acevedo M (2020). The management of agricultural waste biomass in the framework of circular economy and bioeconomy: An opportunity for greenhouse agriculture in Southeast Spain. Agronomy.

[31] Duque-Acevedo M (2020). Agricultural waste: Review of the evolution, approaches and perspectives on alternative uses. Glob. Ecol. Conserv..

[32] G. Abbas, M. Hatatah, A. Ali, E. Touti, A. Alshahir, and A. M. Elrashidi, “A Novel Energy Proficient Computing Framework for Green Computing Using Sustainable Energy Sources,” IEEE Access, vol. 11, 126542–126554 (2023).

[33] Saeed, M., *et al*. Agricultural waste biomass energy potential in Pakistan. in *Proceedings of the International Conference held in Shanghai, PR China*. 2015. Leeds.

[34] Shah AV (2021). Municipal solid waste as a sustainable resource for energy production: State-of-the-art review. J. Environ. Chem. Eng..

[35] Kulkarni BN, Anantharama V (2020). Repercussions of COVID-19 pandemic on municipal solid waste management: Challenges and opportunities. Sci. Total Environ..

[36] Magazzino C, Mele M, Schneider N (2020). The relationship between municipal solid waste and greenhouse gas emissions: Evidence from Switzerland. Waste Manag..

[37] Hasan M (2021). Energy recovery from municipal solid waste using pyrolysis technology: A review on current status and developments. Renew. Sustain. Energy Rev..

[38] Vinti G (2021). Municipal solid waste management and adverse health outcomes: A systematic review. Int. J. Environ. Res. Public Health.

[39] Sharma P (2022). Trends in mitigation of industrial waste: Global health hazards, environmental implications and waste derived economy for environmental sustainability. Sci. Total Environ..

[40] Kwon G (2020). A review of recent advancements in utilization of biomass and industrial wastes into engineered biochar. J. Hazardous Mater..

[41] Aschemann-Witzel J (2015). Consumer-related food waste: Causes and potential for action. Sustainability.

[42] Yuan H-W (2021). Potential for reduced water consumption in biorefining of lignocellulosic biomass to bioethanol and biogas. J. Biosci. Bioeng..

[43] de Azevedo AR (2022). Possibilities for the application of agro-industrial wastes in cementitious materials: A brief review of the Brazilian perspective. Cleaner Mater..

[44] Kurniawan TA (2022). Transformation of solid waste management in China: Moving towards sustainability through digitalization-based circular economy. Sustainability.

[45] Jia X (2022). Regional carbon drawdown with enhanced weathering of non-hazardous industrial wastes. Resources Conserv. Recycling.

[46] Yu H (2020). A stochastic network design problem for hazardous waste management. J. Cleaner Product..

[47] Agamuthu P, Barasarathi J (2021). Clinical waste management under COVID-19 scenario in Malaysia. Waste Manag. Res..

[48] Lee C (2019). Toxic Waste and Race in the United States. Race and the Incidence of Environmental Hazards.

[49] Zamparas M (2019). Medical waste management and environmental assessment in the Rio University Hospital, Western Greece. Sustain. Chem. Pharmacy.

[50] Çetinkaya AY, Kuzu SL, Demir A (2020). Medical waste management in a mid-populated Turkish city and development of medical waste prediction model. Environ. Develop. Sustain..

[51] Mehra M (2020). Extraction of collagen from leather waste to develop aluminium based metal matrix composite. Mater. Today Proc..

[52] Danyang L, Qingyin D, Quanyin T (2022). Study on generation prediction and management mechanism of laboratory hazardous waste in Beijing. Chin. J. Environ. Eng..

[53] Khan BA (2019). Healthcare waste management in Asian developing countries: A mini review. Waste Manag. Res..

[54] Iqbal, A., Abdullah, Y., Nizami, A.S., Sultan, I.A., Sharif, F. Assessment of solid waste management system in Pakistan and sustainable model from environmental and economic perspective. *Sustainability* **14**, 12680. 10.3390/su141912680 (2022).

[55] Chen DM-C (2020). The world’s growing municipal solid waste: Trends and impacts. Environ. Res. Lett..

[56] Jamro IA (2022). Management of university solid waste in China through gasification technology: An analysis of waste composition and energy potential. Environ. Sci. Pollut. Res..

[57] Olalo KF, Nakatani J, Fujita T (2022). Optimal Process network for integrated solid waste management in Davao City, Philippines. Sustainability.

[58] Ibikunle RA (2021). Investigating municipal solid waste generation and management in Ilorin for possible integrated waste-management system. J. Mater. Cycles Waste Manag..

[59] Pudcha T, Phongphiphat A, Towprayoon S (2023). Greenhouse gas mitigation and energy production potentials from municipal solid waste management in Thailand through 2050. Earth Syst. Environ..

[60] Negri C (2020). Anaerobic digestion of food waste for bio-energy production in China and Southeast Asia: A review. Renew. Sustain. Energy Rev..

[61] Namlis K-G, Komilis D (2019). Influence of four socioeconomic indices and the impact of economic crisis on solid waste generation in Europe. Waste Manag..

[62] Singh A (2019). Managing the uncertainty problems of municipal solid waste disposal. J. Environ. Manag..

[63] Yousefi M (2021). Municipal solid waste management during COVID-19 pandemic: Effects and repercussions. Environ. Sci. Pollut. Res..

[64] Ramos A, Rouboa A (2020). Renewable energy from solid waste: Life cycle analysis and social welfare. Environ. Impact Assess. Rev..

[65] A. Ali *et al.*, “A bi-level techno-economic optimal reactive power dispatch considering wind and solar power integration,” IEEE Access, vol. 11, pp. 62799–62819, 10.1109/access.2023.3286930 (2023).

[66] Rashid MI, Shahzad K (2021). Food waste recycling for compost production and its economic and environmental assessment as circular economy indicators of solid waste management. J. Clean. Product..

[67] Yaman C (2020). Investigation of greenhouse gas emissions and energy recovery potential from municipal solid waste management practices. Environ. Develop..

[68] Colvero DA (2020). Economic analysis of a shared municipal solid waste management facility in a metropolitan region. Waste Manag..

[69] Singh E (2022). Solid waste management during COVID-19 pandemic: Recovery techniques and responses. Chemosphere.

[70] Mancini SD (2021). Circular economy and solid waste management: Challenges and opportunities in Brazil. Circ. Econ. Sustain..

[71] Dianati K (2021). A system dynamics-based scenario analysis of residential solid waste management in Kisumu, Kenya. Sci. Total Environ..

[72] Alam P (2022). Energy generation and revenue potential from municipal solid waste using system dynamic approach. Chemosphere.

[73] Mohan S, Joseph CP (2021). Potential hazards due to municipal solid waste open dumping in India. J. Indian Inst. Sci..

[74] Jayaweera M (2019). Management of municipal solid waste open dumps immediately after the collapse: An integrated approach from Meethotamulla open dump, Sri Lanka. Waste Manag..

[75] Nagpure AS (2019). Assessment of quantity and composition of illegal dumped municipal solid waste (MSW) in Delhi. Resources Conserv. Recycling.

[76] Shahabuddin M (2020). A review on the production of renewable aviation fuels from the gasification of biomass and residual wastes. Bioresource Technol..

[77] Indrawan N (2020). Distributed power generation via gasification of biomass and municipal solid waste: A review. J. Energy Inst..

[78] Sun Y (2021). Techno-environmental-economic evaluation on municipal solid waste (MSW) to power/fuel by gasification-based and incineration-based routes. J. Environ. Chem. Eng..

[79] Zhang, Y., *et al*. Gasification technologies and their energy potentials. in *Sustainable Resource Recovery and Zero Waste Approaches*, 193–206. (Elsevier, 2019).

[80] Gao N (2022). Critical assessment of plasma tar reforming during biomass gasification: A review on advancement in plasma technology. J. Hazardous Mater..

[81] Raza MA, Khatri KL, Hussain A (2022). Transition from fossilized to defossilized energy system in Pakistan. Renew. Energy.

[82] Kirby M, Ahmad M-u-D (2022). Can Pakistan achieve sustainable water security? Climate change, population growth and development impacts to 2100. Sustain. Sci..

[83] Rehman E, Rehman S (2022). Modeling the nexus between carbon emissions, urbanization, population growth, energy consumption, and economic development in Asia: Evidence from grey relational analysis. Energy Rep..

[84] Ali, A., Abbas, G., Keerio, M. U., Mirsaeidi, S., Alshahr, S., and Alshahir, A. “Multi-objective optimal siting and sizing of distributed generators and shunt capacitors considering the effect of voltage-dependent nonlinear load models,” IEEE Access, vol. 11, pp. 21465–21487 10.1109/access.2023.3250760 (2023).

[85] Ahmad US (2022). Determinants of renewable energy sources in Pakistan: An overview. Environ. Sci. Pollut. Res..

[86] Khan, N. *et al.*, “A deep learning technique Alexnet to detect electricity theft in smart grids,” *Front. Energy Res.*, vol. 11, p. 1287413, (2023).

[87] Raza MA (2022). Towards achieving 100% renewable energy supply for sustainable climate change in Pakistan. Sustainability.

[88] Mirjat NH (2018). Long-term electricity demand forecast and supply side scenarios for Pakistan (2015–2050): A LEAP model application for policy analysis. Energy.

[89] Habib, S., Abbas, G., Jumani, T. A., Bhutto, A. A., Mirsaeidi, S. & Ahmed, E. M. “Improved whale optimization algorithm for transient response, robustness, and stability enhancement of an automatic voltage regulator system,” *Energies*, **15**(14), 5037 10.3390/en15145037 (2022).

[90] Li H (2022). Physiochemical properties, heavy metal leaching characteristics and reutilization evaluations of solid ashes from municipal solid waste incinerator plants. Waste Manag..

[91] Komilis D (2012). Revisiting the elemental composition and the calorific value of the organic fraction of municipal solid wastes. Waste Manag..

[92] Menikpura S, Basnayake B (2009). New applications of ‘Hess Law’and comparisons with models for determining calorific values of municipal solid wastes in the Sri Lankan context. Renew. Energy.

[93] Holechek JL (2022). A global assessment: Can renewable energy replace fossil fuels by 2050?. Sustainability.

[94] Zhao N, You F (2021). New York State's 100% renewable electricity transition planning under uncertainty using a data-driven multistage adaptive robust optimization approach with machine-learning. Adv. Appl. Energy.

[95] Zhao N, You F (2020). Can renewable generation, energy storage and energy efficient technologies enable carbon neutral energy transition?. Appl. Energy.

[96] Yuan X-C (2018). China's energy transition strategy at the city level: The role of renewable energy. J. Clean. Product..

[97] Yazdanpanah M, Komendantova N, Ardestani RS (2015). Governance of energy transition in Iran: Investigating public acceptance and willingness to use renewable energy sources through socio-psychological model. Renew. Sustain. Energy Rev..

[98] Winkler B (2018). Transition towards renewable energy production? Potential in smallholder agricultural systems in West Bengal, India. Sustainability.

[99] Wiehe J (2021). Nothing to regret: Reconciling renewable energies with human wellbeing and nature in the German Energy Transition. Int. J. Energy Res..

[100] Vidal-Amaro JJ, Sheinbaum-Pardo C (2018). A transition strategy from fossil fuels to renewable energy sources in the Mexican electricity system. J. Sustain. Develop. Energy Water Environ. Syst..

[101] Trencher G, van der Heijden J (2019). Contradictory but also complementary: National and local imaginaries in Japan and Fukushima around transitions to hydrogen and renewables. Energy Res. Social Sci..

[102] Sutherland L-A, Peter S, Zagata L (2015). Conceptualising multi-regime interactions: The role of the agriculture sector in renewable energy transitions. Res. Policy.

[103] Ringkjøb H-K, Haugan PM, Nybø A (2020). Transitioning remote Arctic settlements to renewable energy systems–A modelling study of Longyearbyen, Svalbard. Appl. Energy.

[104] Khan M (2023). Modeling of intelligent controllers for solar photovoltaic system under varying irradiation condition. Front. Energy Res..

